# Computational analysis reveals a correlation of exon-skipping events with splicing, transcription and epigenetic factors

**DOI:** 10.1093/nar/gkt1338

**Published:** 2013-12-24

**Authors:** Zhenqing Ye, Zhong Chen, Xun Lan, Stephen Hara, Benjamin Sunkel, Tim H.-M. Huang, Laura Elnitski, Qianben Wang, Victor X. Jin

**Affiliations:** ^1^Departments of Molecular Medicine, University of Texas Health Science Center at San Antonio, San Antonio, TX 78229, ^2^Department of Molecular and Cellular Biochemistry and the Comprehensive Cancer Center, The Ohio State University, Columbus, OH 43210, USA, ^3^Department of Biomedical Informatics, The Ohio State University, Columbus, OH 43210, USA ^4^Genome Technology Branch, National Human Genome Research Institute, National Institutes of Health, Rockville, MD 20852, USA and ^5^Deparment of Epidemiology and Biostatistics, University of Texas Health Science Center at San Antonio, San Antonio, TX 78229, USA

## Abstract

Alternative splicing (AS), in higher eukaryotes, is one of the mechanisms of post-transcriptional regulation that generate multiple transcripts from the same gene. One particular mode of AS is the skipping event where an exon may be alternatively excluded or constitutively included in the resulting mature mRNA. Both transcript isoforms from this skipping event site, i.e*.* in which the exon is either included (inclusion isoform) or excluded (skipping isoform), are typically present in one cell, and maintain a subtle balance that is vital to cellular function and dynamics. However, how the prevailing conditions dictate which isoform is expressed and what biological factors might influence the regulation of this process remain areas requiring further exploration. In this study, we have developed a novel computational method, graph-based exon-skipping scanner (GESS), for *de novo* detection of skipping event sites from raw RNA-seq reads without prior knowledge of gene annotations, as well as for determining the dominant isoform generated from such sites. We have applied our method to publicly available RNA-seq data in GM12878 and K562 cells from the ENCODE consortium and experimentally validated several skipping site predictions by RT-PCR. Furthermore, we integrated other sequencing-based genomic data to investigate the impact of splicing activities, transcription factors (TFs) and epigenetic histone modifications on splicing outcomes. Our computational analysis found that splice sites within the skipping-isoform-dominated group (SIDG) tended to exhibit weaker MaxEntScan-calculated splice site strength around middle, ‘skipping’, exons compared to those in the inclusion-isoform-dominated group (IIDG). We further showed the positional preference pattern of splicing factors, characterized by enrichment in the intronic splice sites immediately bordering middle exons. Finally, our analysis suggested that different epigenetic factors may introduce a variable obstacle in the process of exon–intron boundary establishment leading to skipping events.

## INTRODUCTION

Alternative splicing (AS) refers to various mechanisms of post-transcriptional gene regulation in higher eukaryotes generating many unique transcripts from a single gene-coding region. During transcription, non-protein-coding sequences (introns) in pre-mRNA molecules are excised by the spliceosome machinery and protein-coding exons are joined together to form mature mRNA molecules. AS events result in mRNAs in which exons have been reconnected with variable inclusion, exclusion and ordinal positioning, and therefore greatly increase the diversity of proteins that can be encoded by the genome. In humans, >90% of multi-exon genes undergo AS (1,2). Many studies have highlighted AS as an important mechanism in many cellular development and differentiation events (3), and errors in splicing regulation may lead to disease states such as muscular dystrophies and premature-aging disorders (4).

There are numerous modes of AS, the most common being the exon skipping event. In this mode, the middle exon in a set of three consecutive exons within a gene-coding region may be included in the mature mRNA under some conditions or in particular tissues, and excluded from the mRNA in others (5). Alternative inclusion (inclusion isoform) or exclusion (skipping isoform) of the middle exon can generate protein isoforms with distinct enzymatic activity or allosteric regulation, and differing, even opposing, biological functions (6,7). It is generally recognized that both the skipping isoform and inclusion isoform could be present at the same time in one cellular condition, where the subtle balance between the two isoforms is perhaps vital for maintenance of cellular homeostasis. How distinct cellular conditions define which isoform predominates and how various biological factors influence the regulation of this process remain incompletely understood.

Recently, advances in next-generation sequencing of messenger RNA (RNA-seq) have enabled us to survey gene expression more accurately (8). In addition, it can provide a more precise measurement of distinct transcript expression levels, as RNA-seq allows direct detection of AS events using reads mapped at splice junctions, including novel splicing events without prior annotation information (9). Several computational frameworks have been developed to calculate the ratio of skipping and inclusion isoforms within one cellular condition using either single- or paried-end RNA-seq data. For example, SpliceTrap (10) approaches exon inclusion level estimation as a Bayesian inference problem by enumerating each tri-exon combination generated by shuffling explicitly known, annotated exons. Another widely used tool, MISO (11), is a probabilistic framework that uses information in single- or paired-end RNA-seq data to comprehensively analyze all major types of alternative pre-mRNA processing at either the exon or isoform level. It uses the inferred assignment of reads to annotated isoforms to quantitate the abundance of the underlying set of alternative mRNA isoforms and estimates confidence intervals. However, both methods heavily rely on the gene and exon annotations corresponding to exon-skipping events that are pre-defined within the reference genome. Unfortunately, the current reference genome is very incomplete due to the complexity of the transcriptome, which hinders the comprehensive investigation of isoform identity and abundance using RNA-seq. Novel methods of isoform inference and estimation from firsthand, raw RNA-seq data without prior knowledge of annotation information is desirable.

**Figure 1.**
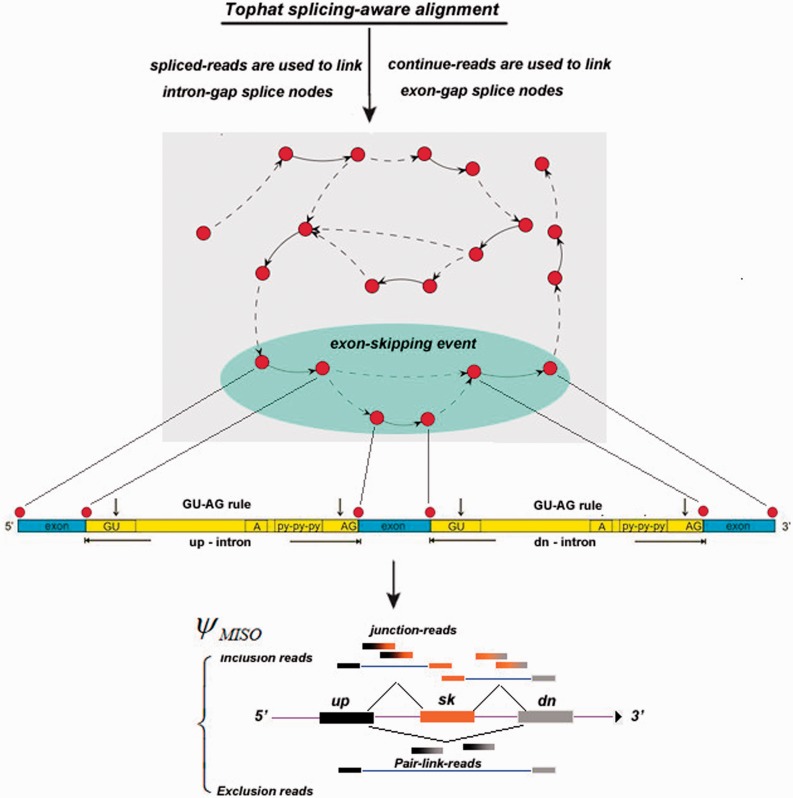
The scheme of the exon-skipping event detection pipeline (GESS). Sequencing reads are aligned to the reference genome using Tophat, a splicing-aware alignment tool. Then the splice-site link graph is created by parsing the alignment, where spliced-reads are used to link the intron-gap splice nodes indicated by the dotted lines, and the remaining reads are used to link the exon-gap splice nodes indicated by the solid lines, since they are generated from an integrated exon without splits. Finally, exon-skipping regions are then identified by iteratively navigating the sub-graph patterns to reveal those corresponding to a skipping event. The detected regions are then compiled for analysis using the MISO model to calculate the *ψ*-value.

Here, we introduce a novel computational method, graph-based exon-skipping scanner (GESS), to detect *de novo* exon-skipping events directly from raw RNA-seq data without prior knowledge of gene-annotation information (detection scheme summarized in Figure 1, also see Materials and methods section for details). First, we build a splicing-site-linking graph from splicing-aware aligned reads using a greedy algorithm. We then iteratively scan this linking graph to obtain those patterns conforming to skipping events. Finally, we apply the MISO model (11) to calculate the ratio of skipping versus inclusion isoforms and determine which is the dominant isoform. We have applied our method to publicly available RNA-seq data in GM12878 and K562 cells from the ENCODE consortium (12) and experimentally validated several skipping site predictions by RT-PCR. Furthermore, we integrated other sequencing-based genomic (‘omics’) data to investigate the impact of splicing activities, TFs and epigenetic histone modifications on splicing outcomes.

## MATERIALS AND METHODS

### A novel computational method to detect *de novo* exon-skipping events

An overview of our computational method for detecting exon-skipping events, GESS, is summarized in Figure 1. Given a set of single- or paired-end RNA-seq reads, we first filter out bad reads with low quality and ambiguous bases. We align the remaining reads to the reference genome (either human hg18/19 or mouse mm8/9/10) using TopHat, a splicing-aware reads-mapping tool (9). After discarding multiply mapped reads, the remaining set of unique aligned reads is composed of two subsets: (i) a set of aligned splicing-reads, which are split between two genomic locations (presumably putative exon–exon junctions), and (ii) a set of aligned constitutive-reads, which are restrictively mapped to a single genomic location (presumably within one exon) rather than splitting between two locations.

Using the two subsets of aligned reads, we build a splice-site-linking graph to identify exon-skipping events for a given transcriptome (Figure 1). To determine splice site locations, we examine the set of spliced-reads one by one, assigning the two genomic positions corresponding to a spliced-read to two individual nodes. We link the two nodes with a dotted-line edge if the number of spliced-reads mapping to these two genomic locations exceeds a defined threshold (default parameter is 8). A dotted-line edge corresponds to an intron gap, and we are able to determine the direction of such lines by invoking the ‘GT-AG’ consensus rule for most vertebrate introns. We also calculate the coverage density between two adjacent splice sites using the set of constitutive-reads. If there is a high density of reads between two splice sites, we would link the corresponding nodes with a solid-line edge. This type of edge corresponds to exonic regions. To assign such a solid-line edge for two nodes, we first arrange the splice sites along the chromosome coordinates, and calculate the depth of coverage for each segment between two adjacent splice sites. For each specific segment carrying a robustly higher signal ratio (i.e. 3.0) relative to the flanking background segments, a solid edge is introduced as an exon gap. Thus, a complex graph is obtained with intronic or exonic links between each pair of splice sites. We then implement a walking strategy on this graph by iteratively navigating each sub-graph to reveal patterns indicative of an exon-skipping event. As shown in Figure 1 (the oval shape), for each combination of six connected nodes (splice sites), we identify instances in which sub-graphs reveal both a tri-exon pattern with three solid-line edges as well as a pattern representative of an exon-skipping event in which the downstream and upstream exons are connected by a dotted-line edge. If no such pattern is identified, we ignore this set of six nodes and move to the next combination. Under this scheme, we are able to identify exon-skipping-event locations and reveal instances of two mutually exclusive splicing modes within such locations, while other types of AS, such as intron retention, are filtered out. We thus define sub-graphs retained by our method as exon-skipping events with two possible exon combination outcomes: the inclusion combination (termed the ‘inclusion isoform’ in which the middle exon is present), and the skipping combination (termed the ‘skipping isoform’ in which the middle exon is excluded). We finally integrate the MISO model (11) to calculate the ratios of the two isoforms for each exon-skipping event and determine which isoform is dominant in each cell model. GESS is available for download at http://motif.bmi.ohio-state.edu/GESS_Web/.

### Evaluating the 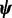-value for exon-skipping events utilizing the MISO model

Although both isoforms are expressed, it is vital to determine which specific isoform predominates. As shown in Figure 1, the inclusion isoform is represented by spliced-reads partially containing the bases of the middle, ‘skipping’, exon (inclusion reads), while reads mapped to the boundary of the upstream- and downstream-exon would represent an alternative-splicing event (exclusion reads). By comparing the reads mapped to the two different isoforms, we are able to calculate the percent of the skipping isoform versus the inclusion isoform. Thus, we utilize the MISO model (11) by introducing the *ψ* parameter to calculate the ratio of two isoforms. By modeling the generative process by which reads are produced from distinct mRNA isoforms in RNA-seq data, the MISO model uses Bayesian inference to compute the probability that a read originated from a particular isoform. MISO uses the inferred assignment of reads to each isoform to quantitate the abundances of the underlying set of alternative mRNA isoforms. Briefly, it can be modeled as the following formulas in the two-isoform case.

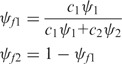

Here, *ψ_k_* denotes the fraction of mRNAs corresponding to the *k*-th isoform, while *c_k_* is the rescaling parameter. And *ψ_f_*_1_ and *ψ_f_*_2_ corresponds, respectively, to the rescaled abundance of the inclusion isoform and skipping isoform. As a convention (in this study), a value *ψ* = ψ*_f_*_1_ ≥ 0.7, indicates that the inclusion isoform is predominantly expressed at an exon-skipping event site, and we refer to these instances collectively as the *ψ*_big_ group. Similarly, a value *ψ* = *ψ_f_*_1_ ≤ 0.3, indicates that the skipping isoform predominates at a particular positions, and we refer to these events as the *ψ*_sml_ group.

### Competition analysis among splice donor and acceptor sites

The analysis of competition between splice donor sites (5′-ss) and acceptor sites (3′-ss) was performed using the MaxEntScan tool, which can calculate the strength of these splicing sites by imposing the MaxEnt framework (13). This model assigns a log-odds ratio (MaxEnt score) to a 9-mer (5′-ss) or a 23-mer (3′-ss) sequence to evaluate the potential that a site would be spliced. For each group (*ψ*_sml_ and *ψ*_big_), we calculated the MaxEnt score for four spanning splice sites within each tri-exon set: upI_ss5 and upI_ss3 (located near the 5′- and 3′-ends of the upstream-intron, respectively), as well as dnI_ss5 and dnI_ss3 (located near the 5′- and 3′-ends of the downstream-intron, respectively). The statistical survey of differential strength among these sites was performed using the Mann–Whitney test.

In addition, we define a pure competitive strength, *S*, derived from the above MaxEnt scores as the following:

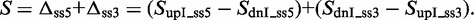

The first term, Δ_ss5_, measures the difference strength of the potential site-pairing, indicating the two donor sites (upI_ss5 and dnI_ss5) competitively connected to the same acceptor site (dnI_ss3). Conversely, the second term, Δ_ss3_, indicates the two acceptor sites (dnI_ss3 and upI_ss3) competitively connected to the same donor site (upI_ss5). Thus *S* can virtually indicate the net effectiveness of competition among the four splice sites at a skipping event location, where larger scores reflect a higher priority for pairing upI_ss5 and dnI_ss3, leading to the skipping isoform.

### Motif analysis of splicing factors

We performed a motif analysis of splicing factors (SFs), which are frequently required for AS regulation. To predict SF-binding sites (SFBS), we used the SFmap software with default settings (14), which is available online at http://sfmap.technion.ac.il. Briefly, SFmap implements the COS(WR) algorithm, which computes similarity scores for a given regulator motif on the basis of information derived from its sequence environment and its evolutionary conservation (15). In this study, we have split the nucleotide sequence involved in each individual exon-skipping event into eight segments, which start from each splicing site and extend 50 bp into the exon and 100 bp into the intron. For each segment, we calculate the *P*-value using a non-parametric Mann–Whitney test between the two groups (*ψ*_sml_ versus *ψ*_big_). A heat map is plotted by transforming the *P*-value to a color scheme. Here, the negative flag along the color bar means the corresponding factor is enriched in the inclusion isoform group (*ψ*_big_), while the positive flag means it is enriched in the skipping isoform group (*ψ*_sml_).

### Distribution of TFs along skipping event sites

ChIP-seq data sets of 44 TFs in K562 cells were downloaded from the ENCODE website. The read densities of these TFs at the eight previously described splice site-spanning regions of both groups (*ψ*_sml_ and *ψ*_big_) were measured. For each TF, the density signals were then transformed to *Z*-scores in both groups, and a heat map was plotted to compare the tag densities between the inclusion and skipping isoform groups. The Pearson correlation co-efficient (*r*) of the *Z*-scores between the two groups for each TF was used as a measure of similarity of the TF-distribution pattern between the two groups. TFs are then arranged from top to bottom in ascending order of the absolute value of *r*.

### Distribution of epigenetic marks along skipping event sites

To investigate the relationship between exon skipping events and epigenetic marks, we obtained the ChIP-seq data of two histone post-translational modifications (H3K36me3 and H3K79me2) in K562 cells, which are thought to influence transcriptional elongation. In addition, Pol-II and nucleosome occupancy data were downloaded from the ENCODE Consortium. For the two groups (*ψ*_sml_ and *ψ*_big_), we calculated the average signal of each mark on the eight regions neighboring splice sites involved in skipping events, normalized by the total number of reads and by subtraction of input control signals.

### Experimental validation of GESS predictions by RT-PCR

RT-PCR was performed as previously described (16) in K562 cells. Briefly, total RNA was isolated using an RNeasy kit (Qiagen, Valencia, CA). cDNA was reverse transcribed from total RNA (2 mg) using a High Capacity cDNA Reverse Transcription Kit (Applied Biosystems, Foster City, CA). The PCR primers for amplifying cDNA fragments between the upstream exon and downstream exon of three skipping event sites are as follows.

- The first skipping event site, within the HPS1 gene.
  5′-GAAGCTCTCGGACACCTACA-3′ (upstream exon)
  5′-GTAGGTCCACAGCAGGCTC-3′ (downstream exon)
- The second skipping event site, within the KEL gene.
  5′-ACCAAAGTGAGGAAGAGCCG-3′ (upstream exon)
  5′-CACAGATGTCTCACAGGGGC-3′ (downstream exon)
- The third skipping-event site, within the FANCD2 gene.
  5′-ATTCCTGCAGTGAGCAGTCT-3′ (upstream exon)
  5′-CAATCCCATCCTGAGTGTCGT-3′ (downstream exon)

PCR was performed with the AccuPrime^TM^ Taq DNA Polymerase System (Invitrogen), and the cycling conditions were: 94°C for 1 min, then 32 cycles of 94°C for 15 s, 58°C for 30 s, 68°C for 30 s.

## RESULTS

### Identification of the exon-skipping events in K562 and GM12878 cells

**Figure 2.**
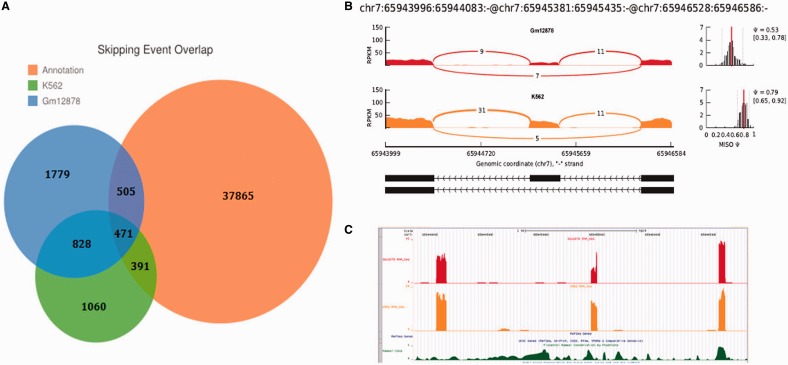
(**A**) A Venn diagram showing an overlapping comparison of exon-skipping events identified by GESS with the annotated events from the MISO website. (**B**) An exon-skipping event detected by GESS, in which both isoforms are present in K562 and GM12878 cells. (**C**) No RefGene information for this skipping event was found on the UCSC track (top panel); the coverage along the chromosome is also provided (bottom panel).

To demonstrate its performance and applicability, we applied the GESS method to publicly available RNA-seq data from K562 and GM12878 cells. These two cell lines are ENCODE Tier 1 cell lines with many publicly available ‘omics’ datasets for further analysis available for each. Using GESS, we identified 2750 exon-skipping events in K562 cells and 3583 events in GM12878 cells (Supplemental Files 1 and 2). Of these events, 1299 were common to both cell lines (Figure 2A). Comparing our results to the annotated exon-skipping database for the human genome, which contains 39 232 events and was downloaded from the MISO website (http://genes.mit.edu/burgelab/miso/), we found only ∼30% of our events overlapped previously annotated skipping events, with many unique skipping events being newly detected by our method. We also observed that a large amount of annotated events were not reported by GESS due to absent/low expression signals or splicing links in the RNA-seq data utilized.

To evaluate our predictions, we compared our results to another *de novo* assembled transcriptome from Cufflinks (17). By breaking each skipping event into its individual exon components, we found that out of 7978 exons corresponding to the total 2750 skipping events in K562 cells, 7168 (90.0%) aligned with exons identified by Cufflinks, while out of 10 322 exons corresponding to the total 3583 skipping events in GM12878 cells, 9305 (90.2%) were exactly aligned with those from Cufflinks (Supplementary Files 3 and 4). This demonstrates that exon boundary identification by our program is very reliable and highly consistent with Cufflinks. We next utilized the transcriptome assembly generated by Cufflinks to identify exon-skipping events for comparison with our GESS-predicted events. Within annotated reference gene sites, we examined the layout (i.e. connections, inclusions and exclusions) of exons for unique transcripts originating from these sites, and we scanned the resultant link-graph in a similar manner to the GESS method to identify exon-skipping sites. We detected 2118 skipping events in K562 cells, and 2102 events in GM12878 cells based on the Cufflinks assembly. Of these events, 480 were common to the GESS predictions in K562, and 504 were shared with GESS predictions in GM12878. The main reason for the discrepancy between the GESS and Cufflinks results is that the GESS method is exon-oriented and focuses on local exon composition searching, while Cufflinks utilizes a greedy global optimization strategy in building a complete transcriptome by assembling all aligned reads into a parsimonious set of transcripts (17). Thus, the Cufflinks approach may introduce some bias when used for skipping event prediction, especially in sites where numerous exons are involved.

**Figure 3.**
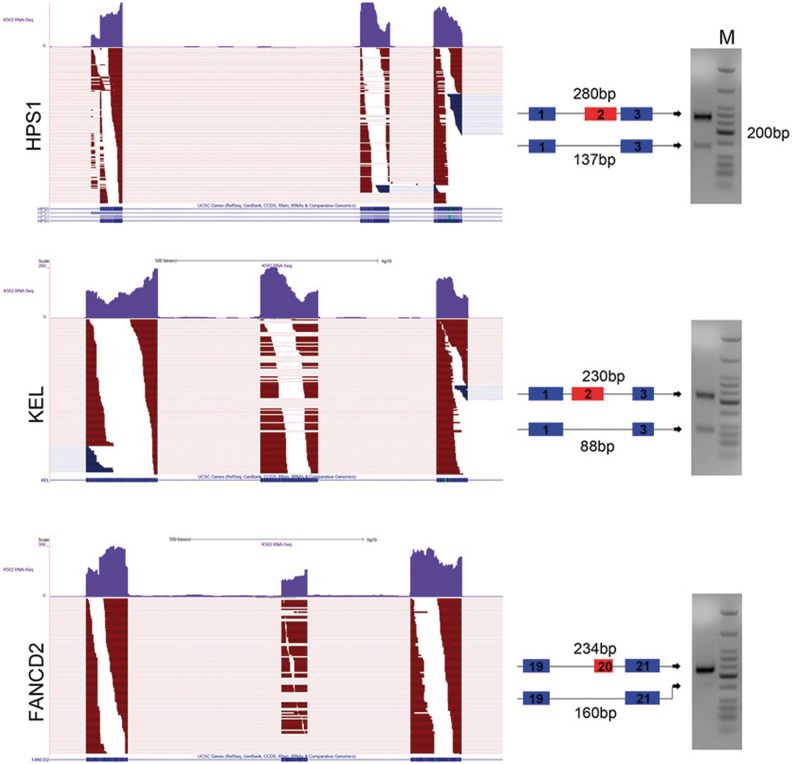
RT-PCR validation of three skipping-event sites predicted by GESS. The mapped reads are partially displayed on the left panel for illustration, with the UCSC gene annotation and Cufflinks transcripts also shown at the bottom. The two DNA bands with indicative fragment sizes are presented on the right panel.

By comparing GESS-predicted skipping events with the annotated RefSeq database (UCSC HG19 RefSeq), in which each exon-skipping event can be mapped to a specific annotated gene, we found 40 skipping events that were not assignable to any known genes in K562 cells, while 34 events lacked annotations in GM12878 cells. As an example shown in Figure 2B, we observed three adjacent exons on chromosome 7 covered by numerous reads in which the alignment pattern of splicing-reads revealed two isoforms with differential expression ratios in the two cell lines. However, no gene annotation information exists for this genomic region (see the RefSeq gene track in Figure 2C) and no skipping event annotation can be found in the MISO dataset. We chose several skipping event sites from the GESS predictions for experimental validation by RT-PCR. We restricted our selection to sites with a higher *ψ*-value ∼0.8, and ensured that both the skipping and inclusion isoforms would have moderate, detectable expression levels. Among 26 potential sites, we chose three novel exon-skipping sites based on the strict criteria that they were not predicted by Cufflinks and no AS annotations were reported in RefGene. Figure 3 shows that, using PCR primers specific to the upstream- and downstream-most exons, all the three skipping-event sites produced two distinct DNA bands with sizes corresponding to the inclusion and skipping isoforms. Importantly, predictions from Cufflinks and annotations in UCSC RefGene present either the inclusion or skipping isoform in these locations only.

**Figure 4.**
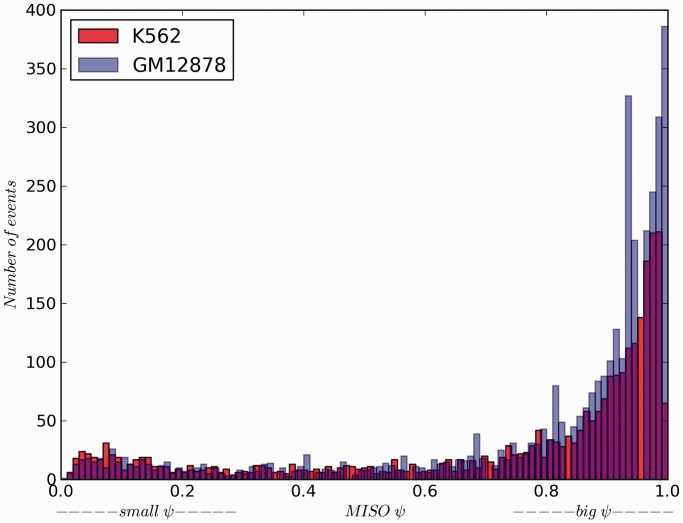
The distribution of *ψ*-values in K562 (purple) and GM12878 cells (red). We defined two groups according to the smaller values (≤0.3, SIDG) and bigger values (≥0.7, IIDG).

We further calculated the *ψ*-value for each exon-skipping event identified by our method for both cell lines using MISO. We found that 387 of the 2750 events in K562 cells had a *ψ*-value <0.3, and we assigned such transcripts to the skipping-isoform-dominated group (SIDG, or indicated by *ψ*_sml_), while the 1993 events with a *ψ*-value > 0.7 were assigned to the inclusion-isoform-dominated group (IIDG or indicated by *ψ*_big_) (Figure 4). For GM12878 cells, 339 out of the 3583 exon-skipping events were assigned to SIDG, and 2832 events were assigned to IIDG. Our results showed that although there are thousands of skipping events in a particular cell type, most exon-skipping event sites preferentially express the inclusion isoform.

### Competition analysis between donor and acceptor splicing sites

**Figure 5.**
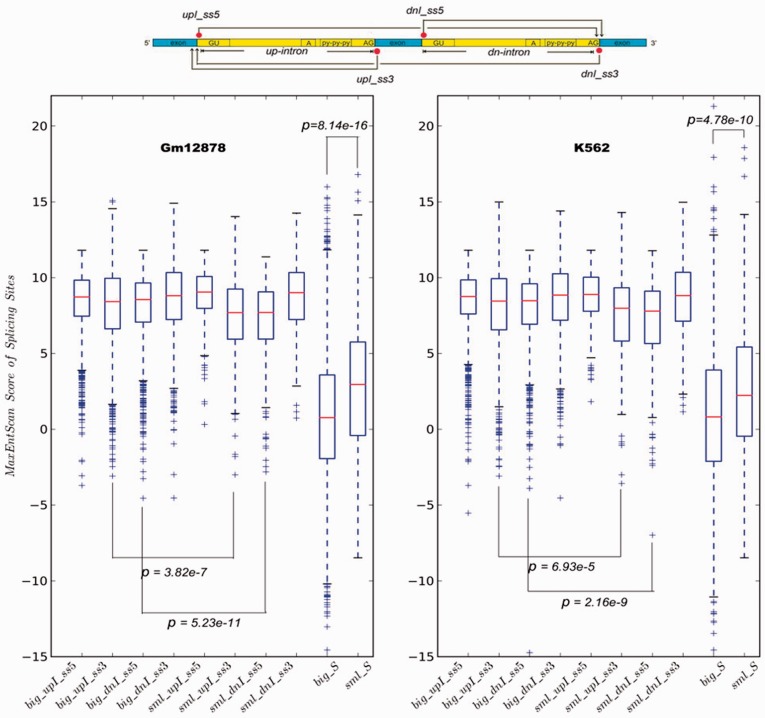
A plot showing the distribution of splice site strengths of the four splice sites (upI_ss5: upstream intron donor site; upI_ss3: upstream intron acceptor site; dnI_ss5: downstream intron donor site; dnI_ss3: downstream intron acceptor site) involved in skipping events for the two groups (*ψ*_big_ and *ψ*_sml_) calculated by MaxEntScan. The two right-most plots in each panel represent the pure competition scores forupI_ss5 and dnI_ss3 in both groups. *P*-values were calculated by a Mann–Whitney test.

It is well known that there is an intense competition between splicing donor and acceptor sites (5′-ss and 3′-ss, respectively) involved in the skipping events. As shown in Figure 5, the acceptor sites on the upstream intron (upI_ss3) and the downstream intron (dnI_ss3) will competitively connect to the donor site on the upstream intron (upI_ss5); similarly, the donor sites on the downstream intron (dnI_ss5) and the upstream intron (upI_ss5) will competitively approach the acceptor site on the downstream intron (dnI_ss3). Splice sites can be classified as ‘weak’ or ‘strong’ according to their similarity to consensus motifs, with stronger splice sites being utilized preferentially. In order to estimate the strengths of the splice sites in the SIDG versus IIDG sets, we used the MaxEntScan tool (13). This model will assign a log-odds ratio (MaxENT score) to a 9-mer (the 5′-splice donor site) and a 23-mer (the 3′-splice acceptor site) sequence. The higher the score, the higher likelihood of the given splice site being used by the splicing machinery to form the mature transcript.

We found that the mean scores for the two splice sites bordering the middle, ‘skipping’, exon (dnI-ss5 and upI-ss3) were smaller in the *ψ*_sml_ group compared to scores in the *ψ*_big_ group in GM12878 cells (Table 1 and Figure 5), with the *P*-values 5.23*e* – 11 and 3.82*e* – 7, respectively. A similar result is obtained in K562 cells. These results indicate that at exon-skipping event sites, skipping isoforms predominate in cases where the splice sites bordering the middle exon exhibit relatively weak splice site strength, resulting in excision of the middle exon and the flanking introns as a single unit. Conversely, stronger splice site strength at the edges of the middle exon can be found in skipping event sites where the inclusion isoform predominates, allowing for more efficient utilization of these splice sites and leading to the removal of the flanking introns individually.

**Table 1.** The MaxEnt scores for the splice sites involved in exon-skipping events

		upI_ss5	upI_ss3	dnI_ss5	dnI_ss3
K562	*ψ*_big_	8.4441 (2.0326)	8.1470 (2.5978)	7.9882 (2.2498)	8.6269 (2.4195)
	*Ψ*_sml_	8.6726 (1.6837)	7.5126 (3.0301)	7.0432 (3.0244)	8.5711 (2.3560)
GM12878	*ψ*_big_	8.4380 (1.9206)	8.1967 (2.5586)	8.1299 (2.0937)	8.6975 (2.3073)
	*Ψ*_sml_	8.8119 (1.7254)	7.4730 (2.6068)	7.1737 (2.6686)	8.7606 (2.1912)

We also conducted an analysis of the pure competitive strength of splice sites within exon-skipping event locations for both cell types. We calculated *S* for each individual event in the *ψ*_sml_ and *ψ*_big_ groups, where higher *S*-values reflect a higher priority for pairing upI_ss5 and dnI_ss3, leading to the skipping isoform. A *t*-test revealed that the *ψ*_sml_ group has a significantly larger *S*-value than the *ψ*_big_ group in both GM12878 cells (*P* = 8.14*E* – 16) and K562 cells (*P* = 4.78*E* – 10) (two right-most plots in each panel of Figure 5). This result further supported a notion that those splice sites (upI_ss3 and dnI_ss5) involved in the middle exon inclusion activity in *ψ*_big_ group, are indeed competitively stronger strength than those presented in *ψ*_sml_ group.

### Motifs analysis of SFs involved in exon-skipping events

Differential RNA splicing is controlled by a system in which *trans*-acting proteins bind to *cis*-acting elements (functional sequence motifs) on pre-mRNA molecules. *Cis*-acting elements can be generally classified into intronic-splicing enhancers (ISEs) or silencers (ISSs) and exonic-splicing enhancers (ESEs) or silencers (ESSs). These elements usually locate near splicing sites and act positively or negatively on the selection of alternative splice sites (5,18). The *trans*-acting pre-mRNA-binding proteins, often called SFs, include splicing activators that promote the usage of a particular splice site and splicing repressors that reduce the usage of a particular site.

**Figure 6.**
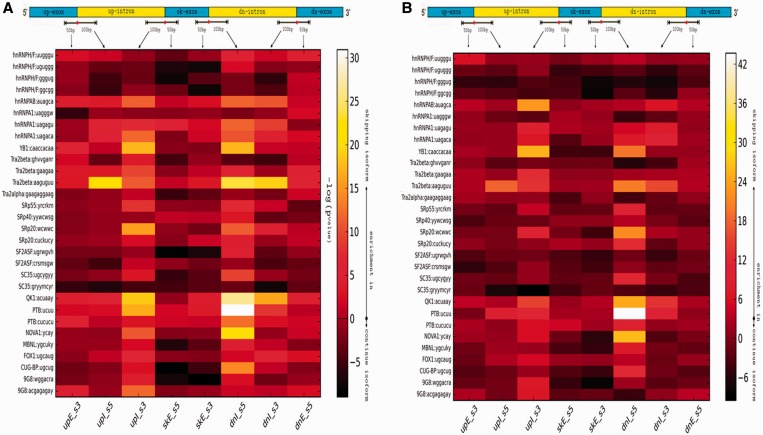
A heat map showing an enrichment of similarity scores predicted by SFMap for SF motifs in eight exon-skipping regions in K562 (**A**) and GM12878 (**B**) cells, respectively. The value in the heat map is the –log_10_(*P*-value) of a series of Mann–Whitney tests comparing the normalized density of SFBS predictions in SIDG versus IIDG.

We examined how these ESE’s and ISEs might distribute around splice sites within SIDG and IIDG exon-skipping-event locations based on identification of their recognition motifs, and asked how their positional patterns could affect splicing outcomes. We scanned for SF motifs in the sequences spanning splice sites (50 bp extending into the exon and 100 bp extending into the intron) within skipping event locations using the SFMap tool, which is designed to map SFBS in human genomic regions using the COS(WR) algorithm (14,15). We then compared the frequency of predicted SFBSs on the eight defined regions immediately adjacent to splice sites in SIDG versus IIDG locations. As shown in Figure 6, a majority of these SFs’ motifs are highly enriched in the intronic regions immediately bordering the middle, skipping exon (upI_ss3 and dnI_ss5) compared with the upstream and downstream exons in both cell types. The splice donor site on the downstream intron (dnI_ss5) has a stronger intensity signal than the splice acceptor site on the upstream intron (upI_ss3). Furthermore, motifs for several known factors involved in AS events, such as PTB, NOVA1 and QK1, were identified. Previous studies have shown a strong enrichment of PTB-binding sites in AS event locations (19), indicating its prominent role in regulating AS in addition to its known function in regulating constitutive splicing events (20). A recent study (21) showed that PTB likely interferes with U1 binding to the downstream splice donor site (dnI_ss5) near the middle skipping-exon, prohibiting the process termed ‘intron definition’ for the downstream intron. On the other hand, PTB may inversely promote the larger-scale intron definition of the bigger intron that include the upstream intron, skipped exon and downstream intron together as a whole one unit, leading to an exon-skipping event.

Interestingly, we also observed that two different motifs of the human serine/arginine-rich (SR) protein SC35 seem to have different distributions and enrichment levels in SIDG versus IIDG. One is enriched in the upI_ss3 sites in IIDG involved in exon inclusion, and the other is enriched in the dnI_ss5 sites in SIDG involved in exon exclusion, suggesting that SC35 may have diverse functional roles in splicing processing (22,23).

### Correlation of TFs with exon-skipping events

**Figure 7.**
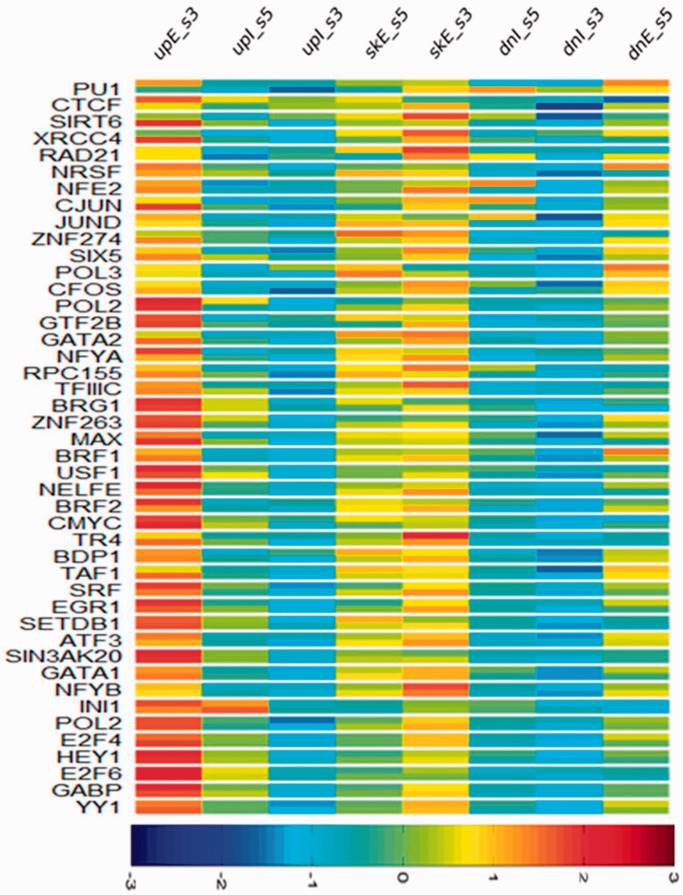
A heat map of *Z*-scores showing a distribution of the tag densities of 44 TFBSs at the eight skipping event regions for both IIDG and SIDG in K562 cells. *Z*-scores were calculated for each of the eight regions for all TFs in both groups. A Pearson correlation coefficient (*r*) of the *Z*-scores between two groups for each TF was used as a measure of similarity of the TF distribution pattern between the two groups, and all TFs are sorted by *r*-values.

Several studies have shown that some TFs could affect splicing outcomes by interplay with the spliceosome complex during transcription (24), thus we wanted to study the correlation between certain TFs and AS events at a genome-wide scale. Since K562 cells have the most ChIP-seq data sets available from the ENCODE website (12), we chose this cell line for this correlation analysis. We collected ChIP-seq datasets for 44 TFs and plotted the distribution of their binding signals along the eight regions surrounding splicing sites within predicted exon-skipping event locations. We found that the signals for most TFs were enriched in exonic regions, and consistent with gene body structure progressing from the promoter to the polyA tail regions, TF signals tended to decrease from the upstream to the middle and finally to the downstream exons (Figure 7). Interestingly, donor sites (upI_ss5 and dnI_ss5) had stronger binding signals than acceptor sites (upI_ss3 and dnI_ss3), which suggests that donor sites may host more regulatory factors and play a more important role in determining splicing outcomes. In addition, we calculated the correlation coefficient (*r*) between the IIDG and SIDG for each TF. We found that most of these TFs have large coefficient values approaching 1.0, implying that there are similar binding patterns between the two groups for these factors. This is consistent with our knowledge that TFs are generally engaged in transcriptional regulation rather than isoform switching regulation (25). However, PU1 and CTCF are exceptional, showing highly differential binding patterns, with *P*_coef_ = 0.1287 and *P*_coef_ = 0.2813, respectively. The differential pattern for PU1 between the two groups suggests that PU1 may regulate the AS process of its target genes, leading to the distinctive erythroid character of K562 cells, derived from a leukemia patient. As for CTCF, an insulator binding protein, a recent study has shown an unforeseen, intragenic role of CTCF that links DNA methylation with AS (26). By alternately binding to and releasing from its target sites, CTCF creates a transient roadblock preventing Pol-II-mediated transcript elongation, leading either to exon inclusion or exclusion. Taken together, our analysis supports the notion that some TFs regulate not only quantitative gene-expression levels, but also isoform switching events during gene expression.

### Correlation of epigenetic marks with exon-skipping events

**Figure 8.**
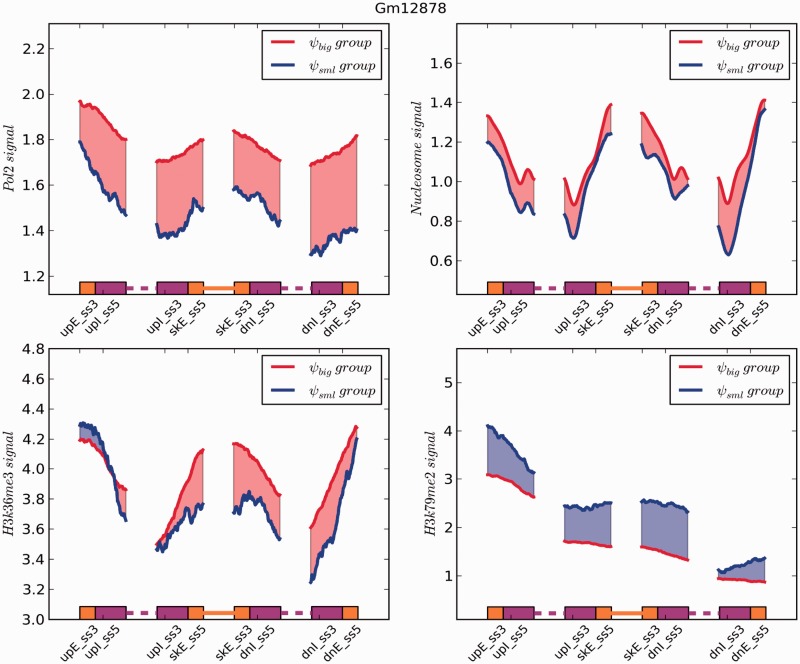
A plot showing the distribution of signal intensities of two histone modifications, Pol-II and nucleosome occupancy on upstream, skipping, and downstream exon–intron boundaries in GM12878 cells. The reads were normalized by the total read number and the exon body length.

**Figure 9.**
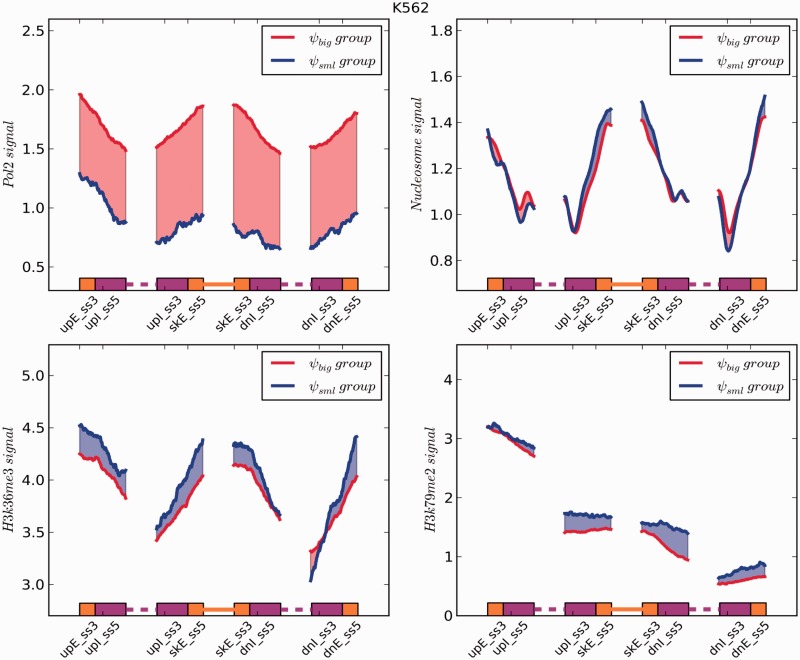
A plot showing the distribution of signal intensities of two histone modifications, Pol-II and nucleosome occupancy on upstream, skipping and downstream exon-intron boundaries in K562 cells. The reads were normalized by the total read number and the exon body length.

Recent studies have shown that different molecular events occur co-transcriptionally and can impact AS, such as chromatin remodeling and histone dynamics (27,28). For example, the speed of RNA polymerase II-mediated transcript elongation and different chromatin states affect splicing outcomes (29). In order to understand the relationship between chromatin modifications and exon skipping events, we analyzed ‘omics’ data for two epigenetic marks associated with transcription elongation, H3K36me3 and H3K79me2, as well as for Pol-II and nucleosome occupancy. We found the signal intensity of Pol-II to be strongly enriched over splice site spanning regions in the *ψ*_big_ group compared to the *ψ*_sml_ group in both cell lines (Figures 8 and 9). For H3K36me3, we observed a difference in enrichment patterns between GM12878 and K562 cells across the *ψ*_big_ and *ψ*_sml_ groups. In K562 cells (Figure 9), this mark showed higher enrichment levels over all splice site spanning regions within the *ψ*_sml_ group. However, in GM12878 cells, this mark shows stronger enrichment across all splice-site spanning regions in the *ψ*_big_ group with the exception of the region from upE_ss3 to upI_ss5. This result indicates that H3K36me3 is not only involved in coupling transcription and splicing events, but also in regulating splicing processes in a cell type- and perhaps gene site-specific manner. For H3K79me2, we observed that it is enriched over splice sites in the *ψ*_sml_ group versus the *ψ*_big_ group in both cell types. Interestingly, with the exception of H3K79me2, the distribution of these transcription and epigenetic factors exhibited decreasing enrichment when progressing from an exon toward an intron, but increasing enrichment when progressing from an intron to an exon. This suggests these factors they may take part in or are sensitive to exon–intron boundary establishment (30). Taken together, our analysis suggests that different epigenetic factors may introduce a variable obstacle in the process of exon–intron boundary establishment leading to skipping events.

## DISCUSSION

Exon-skipping, as a common mode of AS, has been intensively studied lately (5). In this study, we have developed a novel computational method called GESS, to detect both *de novo* and annotated exon-skipping events from RNA-seq data without prior knowledge of gene annotation information. In addition, we demonstrated the performance and applicability of our GESS method using RNA-seq data in K562 and GM12878 cells, two ENCONDE Tier 1 cell lines (see Results section).

A notable advantage of our GESS method is that it is capable of capturing *de novo* exon-skipping events. Since transcriptional regulation in a cell is complex and dynamic, resulting in diverse outcomes under different physiological conditions, many current approaches to skipping event identification rely on annotated exon information. Such approaches may not be able to capture the full landscape of gene expression *in situ*, sometimes leading to errors in interpretation of results. Our GESS method instead builds a splice-site-link graph from first-hand, raw RNA-seq reads and then implements a walking strategy on this graph by iteratively navigating sub-graphs to reveal those with a pattern corresponding to an exon-skipping event. Thus, it can provide a more accurate and thorough picture of skipping events associated with a particular physiological condition within a cell. Furthermore, we integrated the MISO model (11) into our method to determine which isoform, skipping- or inclusion-isoform, is the dominant transcript produced from a skipping-event site, where maintenance of the subtle balance between the two mRNA molecules is vital to cellular function and dynamics.

Although AS events are common in metazoan expression landscapes and important to cellular functions, complexities in accurate transcriptome assembly and the high degree of variability in expression depending upon the prevailing cellular conditions prevent complete exploration of the mechanisms regulating their occurrence. Splicing of mRNA is generally performed by a snRNA and protein complex known as the spliceosome, containing five snRNPs (U1, U2, U4, U5 and U6) and multiple auxiliary proteins. U1 binds to the 5′-GU splice donor site and U2 generally binds to the branch site on the intron with the assistance of the U2AF protein factors (5,31). Based on motif distributions, our analysis reveals that several SFs (including PTB, NOVA1 and QK1) may prefer to bind to the 5′-splice donor sites downstream of the middle, skipped exon in event locations where the skipping-isoform predominates, and in doing so they may modulate the affinity of U1 binding through target site competition. Though the 3′-splice acceptor sites on the upstream intron are also enriched with motifs for several SFs, the signal intensity is relatively lower. As we know, initial splice-site recognition in nascent transcripts is mediated by exon definition interactions, where the binding of splicing components to a 5′-splice site promotes U2AF recognition of the 3′-splice site lying upstream across the exon (32). This leads to the formation of an exon definition complex before assembly of a spliceosome across the intron. Interestingly, for the skipping events, it seems that these specific SFs whose motifs are enriched on the 5′-splice donor sites near the skipping exon may play important roles in AS mechanism. By blocking the intron definition interaction for the downstream intron, these factors may promote the site-pairing interaction between the 5′-splice site on the upstream intron (upI_ss5) and the 3′-splice site on the downstream intron (dnI_ss3) to form a larger defined intronic region, leading to production of a skipping isoform.

Furthermore, TFs may also take part in AS regulation, as several recent studies have demonstrated that some TFs could affect splicing outcomes by interacting with the spliceosome complex during transcription (24). A comprehensive proteomic analysis of the human spliceosome reported that at least 30 out of the 145 spliceosomal proteins have either known or novel rolesin coupling splicing events with other gene expression steps. For example, a study from Zhou *et al.* showed that TFs CA150, XAB2 and SKIP associate with the spliceosome (33). In our analysis we also found that two TFs, PU1 and CTCF, are likely involved in exon-skipping events. PU1 is an important factor involved in malignant leukemia, with a role in splicing activity (34,35). CTCF, known as an insulator binding protein, was recently shown to have an unforeseen role in AS in cooperation with methylation dynamics (26). Interestingly, these two TFs exhibited the most significantly divergent distribution patterns over splice sites in the SIDG versus the IIDG locations in the K562 cells. This result suggests that there is an important crosstalk between these TFs and the spliceosome, impacting splicing outcomes. However, further mechanistic studies are needed to support a splicing regulatory function of PU1 and CTCF.

Chromatin structure, determined by many different epigenetic modifications such as histone modifications and DNA methylation, may play a co-transcriptional role in pre-mRNA processing by influencing RNA polymerase-II (Pol-II)-mediated transcript elongation (24,28,36), as well as in influencing exon and intron definition. In this study, we showed that the enrichment levels for individual histone marks differ over the splice sites involved in skipping events, suggesting that different epigenetic factors may assist or provide an obstacle in the process of exon–intron boundary establishment at skipping event locations.

In summary, our computational analysis not only reveals a correlation of exon-skipping events with splicing, transcription and epigenetic factors, but also provides guidance for biologists to explore further mechanistic studies in the emerging field of chromatin remodeling as it relates to RNA processing.

## SUPPLEMENTARY DATA

Supplementary Data are available at NAR Online.

Supplementary Data

## References

[1] Pan Q, Shai O, Lee LJ, Frey BJ, Blemcowe BJ (2008). Deep surveying of alternative splicing complexity in the human transcriptome by high-throughput sequencing. Nat. Genet..

[2] Wang ET, Sandberg R, Luo S, Khrebtukova I, Zhang L, Mayr C, Kingsmore SF, Schroth GP, Burge CB (2008). Alternative isoform regulation in human tissue transcriptomes. Nature.

[3] Matlin AJ, Clark F, Smith CWJ (2005). Understanding alternative splicing: towards a cellular code. Nat. Rev. Mol. Cell Biol..

[4] Tazi J, Bakkour N, Stamm S (2009). Alternative splicing and disease. Biochim. Biophys. Acta..

[5] Black DL (2003). Mechanisms of alternative pre-messenger RNA splicing. Ann. Rev. Biochem..

[6] Christofk HR, Vander Heiden MG, Wu N, Asara JM, Cantley LC (2008). Pyruvate kinase M2 is a phosphotyrosine-binding protein. Nature.

[7] Cooper TA, Wan L, Dreyfuss G (2009). RNA and disease. Cell.

[8] Garber M, Grabherr MG, Guttman M, Trapnell C (2011). Computational methods for transcriptome annotation and quantification using RNA-seq. Nat. Methods.

[9] Trapnell C, Pachter L, Salzberg S (2009). TopHat: discovering splice junctions with RNA-Seq. Bioinformatics.

[10] Wu J, Akerman M, Sun S, McCombie WR, Krainer AR, Zhang MQ (2011). SpliceTrap: a method to quantify alternative splicing under single cellular conditions. Bioinformatics.

[11] Katz Y, Wang ET, Airoldi EM, Burge CB (2010). Analysis and design of RNA sequencing experiments for identifying isoform regulation. Nat. Methods.

[12] The ENCODE Project Consortium (2012). An integrated encyclopedia of DNA elements in the human genome. Nature.

[13] Yeo G, Burge CB (2004). Maximum entropy modeling of short sequence motifs with applications to RNA splicing signals. J. Comput. Biol..

[14] Paz I, Akerman M, Dror I, Kosti I, Mandel-Gutfreund Y (2010). SFmap: a web server for motif analysis and prediction of splicing factor binding sites. Nucleic Acids Res..

[15] Akerman M, David-Eden H, Pinter RY, Mandel-Gutfreund Y (2009). A computational approach for genome-wide mapping of splicing factor binding sites. Genome. Biol..

[16] Chen Z, Zhang C, Wu D, Chen H, Rorick A, Zhang X, Wang Q (2011). Phospho-MED1-enhanced UBE2C locus looping drives castration-resistant prostate cancer growth. EMBO J..

[17] Trapnell C, Williams BA, Pertea G, Mortazavi AM, Kwan G, van Baren MJ, Salzberg SL, Wold B, Pachter L (2010). Transcript assembly and quantification by RNA-Seq reveals unannotated transcripts and isoform switching during cell differentiation. Nat. Biotech..

[18] Chen M, Manley JL (2009). Mechanisms of alternative splicing regulation: insights from molecular and genomics approaches. Nat. Rev. Mol. Cell Biol..

[19] Spellman R, Smith CW (2006). Novel modes of splicing repression by PTB. Trends Biochem. Sci..

[20] Xue Y, Zhou Y, Wu T, Zhu T, Ji X, Kwon YS, Zhang C, Yeo G, Black DL, Sun H (2009). Genome-wide analysis of PTB-RNA interactions reveals a strategy used by the general splicing repressor to modulate exon inclusion or skipping. Mol. Cell.

[21] Sharma S, Maris C, Allain FH, Black DL (2011). U1 snRNA directly interacts with polypyrimidine tract-binding protein during splicing repression. Mol. Cell.

[22] Lin S, Coutinho-Mansfield G, Wang D, Pandit S, Fu XD (2008). The splicing factor SC35 has an active role in transcriptional elongation. Nat. Struct. Mol. Biol..

[23] Tripathi K, Parnaik VK (2008). Differential dynamics of splicing factor SC35 during the cell cycle. J. Biosci..

[24] Kornblihtt AR, de la Mata M, Fededa JP, Munoz MJ, Nogues G (2004). Multiple links between transcription and splicing. RNA.

[25] Kim HD, O’Shea EK (2008). A quantitative model of transcription factor-activated gene expression. Nat. Struct. Mol. Biol..

[26] Shukla S, Kavak E, Gregory M, Imashimizu M, Shutinoski B, Kashlev M, Oberdoerffer P, Sandberg R, Oberdoerffer S (2011). CTCF-promoted RNA polymerase II pausing links DNA methylation to splicing. Nature.

[27] Luco RF, Pan Q, Tominaga K, Blencowe BJ, Pereira-Smith OM, Misteli T (2010). Regulation of alternative splicing by histone modifications. Science.

[28] Luco RF, Allo M, Schor IE, Kornblihtt AR, Misteli T (2011). Epigenetics in alternative pre-mRNA splicing. Cell.

[29] de la Mata M, Alonso CR, Kadener S, Fededa JP, Blaustein M, Pelisch F, Cramer P, Bentley D, Kornblihtt AR (2003). A slow RNA polymerase II affects alternative splicing in vivo. Mol. Cell.

[30] Schwartz S, Meshorer E, Ast G (2009). Chromatin organization marks exon-intro structure. Nat. Struct. Mol. Biol..

[31] Wahl MC, Will CL, Luhrmann R (2009). The spliceosome: design principles of a dynamic RNP machine. Cell.

[32] Sharma S, Kohlstaedt LA, Damianov A, Rio DC, Black DL (2008). Polypyrimidine tract binding protein controls the transition from exon definition to an intron defined spliceosome. Nat. Struct. Mol. Biol..

[33] Zhou Z, Licklider LJ, Gygi SP, Reed R (2002). Comprehensive proteomic analysis of the human spliceosome. Nature.

[34] Guillouf C, Gallais I, Moreau-Gachelin F (2006). SPI-1/PU. 1 oncoprotein affects splicing decisions in a promoter-binding dependent manner. J. Biol. Chem..

[35] Gupta P, Gurudutta GU, Verma YK, Kishore V, Gulati S, Sharma RK (2006). PU.1: an ETS family transcription factor that regulates leukemogenesis besides normal hematopoiesis. Stem. Cells Dev..

[36] Jung I, Kim SK, Kim M, Han YM, Sung Kim YS, Kim D, Lee D (2012). H2B monoubiquitylation is a 5′-enriched active transcription mark and correlates with exon-intron structure in human cells. Genome Res..

