# Supplementary material for: Computational analysis reveals a correlation of exon-skipping events with splicing, transcription and epigenetic factors
Source: Nucleic Acids Res. 2013 Dec 24;42(5):2856–69. doi: 10.1093/nar/gkt1338 (PMC3950716; doi:10.1093/nar/gkt1338)
Supplement: Supplementary Data [file supp_42_5_2856__index.html]

Computational analysis reveals a correlation of exon-skipping events with splicing, transcription and epigenetic factors — Supplementary Data 

# Computational analysis reveals a correlation of exon-skipping events with splicing, transcription and epigenetic factors

## Supplementary Data

files

**Files in this Data Supplement:**

- Supplementary Data - xls file
- Supplementary Data - xls file
- Supplementary Data - xls file
- Supplementary Data - xls file
